# A new stiffness-sensing test to measure damage evolution in solids

**DOI:** 10.1038/s41598-021-04452-9

**Published:** 2022-01-10

**Authors:** Yichi Song, Doneill J. Magmanlac, Vito L. Tagarielli

**Affiliations:** Department of Aeronautical Engineering, Imperial College London, London, SW7 2AZ UK

**Keywords:** Characterization and analytical techniques, Mechanical properties

## Abstract

We propose and assess a procedure to measure the damage evolution in solids as a function of the applied strain, by conducting stiffness-sensing mechanical tests. These tests consist in superimposing to a monotonically increasing applied strain numerous, low-amplitude unloading/reloading cycles, and extracting the current stiffness of the specimens from the slope of the stress–strain curve in each of the unloading/reloading cycles. The technique is applied to a set of polymeric and metallic solids with a wide range of stiffness, including CFRP laminates loaded through the thickness, epoxy resins, injection-moulded and 3D printed PLA and sintered Ti powders. The tests reveal that, for all the materials tested, damage starts developing at the very early stages of deformation, during what is commonly considered an elastic response. We show that the test method is effective and allows enriching the data extracted from conventional mechanical tests, for potential use in data-driven constitutive models. We also show that the measurements are consistent with the results of acoustic and resistive measurements, and that the method can be used to quantify the viscous response of the materials tested.

## Introduction

The concept of macroscopic damage variables, introduced in early works by Kachanov^1^ and Rabotnov^2^ to describe the macroscopic effect of the microscopic loss of cohesion in solids, has become fundamental in continuum mechanics to formulate constitutive models of solid materials, thanks to the development of Continuum Damage Mechanics (CDM)^3–5^. CDM suggests that fracture is the result of the accumulation of micro-cracks or voids in the material and introduces the concept of effective stress^6^.

Measurements of the onset and evolution of damage in a material as a function of the imposed deformation would be vital to calibrate CDM constitutive models, but such measurements are very difficult to conduct. Consequently, the current practice involves parametrising the damage evolution law and determining the optimal values of the parameters by reverse engineering procedures. This has significant drawbacks, as it involves assuming (i) the damage initiation point, (ii) the shape of the damage evolution curve, and (iii) a characteristic length-scale to compute the traction–separation law. The procedure is further complicated by the inherent mesh-sensitivity encountered when modelling strain-softening of materials.

In the last few years, a growing number of researchers have applied data analytics and machine learning techniques to the constitutive response of solids. In these studies, constitutive models for solids are determined by analysis of datasets obtained from microstructure analysis and stress–strain histories, extracted from either experiments or from detailed micro-scale numerical simulations (e.g.^7–9^). The extension of these approaches to damage models requires information on the evolution of the cohesion of the solid as a function of the applied deformation; this paper proposes a technique to measure such information.

The damage in a solid can be quantified by the degradation of its stiffness, as discussed by Lemaitre and Dufailly^6^ and Bonora et al.^10^, who performed occasional, partial unloading of specimens during uniaxial mechanical testing, measuring the current effective elastic modulus of the specimen *E* and calculating the damage variable *D* as

1 $$D = 1 - \frac{E}{{E_{0} }}$$

where $E_{0}$ is the initial stiffness. While this type of experiment is now very common, to the best of our knowledge no other studies have attempted extending this simple technique to obtain near-continuous measurements of the effective stiffness of the sample, which is what we carefully pursue in this study.

Damage can also be obtained through hardness measurement by indentation tests, as proposed in^6^ and later used in^11–13^; in indentation tests the current stiffness can be obtained from the initial slope of the load–displacement curve upon unloading, as suggested in^14^. In principle, indentation can be applied to a material being probed in different modes, for example in tension; this however requires extensive sample preparation^15^ and interrupting the test being performed in order to conduct the indentation experiments; it also results in very localised damage measurements. Oliver and Pharr^14^ proposed an improved indentation test, referred to as Continuous Stiffness Measurement (CSM), which enabled a near-continuous measurement of the contact stiffness during the loading phase of an indentation test. CSM is performed by superimposing an oscillating force to the monotonically increasing load applied to the indenter. This technique inspires the method developed in the present study, which focuses on tests with uniaxial loading of the specimens.

In their seminal paper Lemaitre and Dufailly^6^ also discuss other indirect damage measurement methods, based on measurements of the speed of sound in the solid (further developed by Boccaccini and Boccaccini^16^) or of the electrical resistance of the specimen^13,17^. In this paper we will present results from acoustic and electrical stiffness-sensing tests, for the purpose of comparing, for a selected material, acoustic and electrical data to the mechanical stiffness-sensing tests.

We propose an extension of the CSM method to uniaxial mechanical tests, resulting in a mechanical, near-continuous stiffness-sensing technique that can be used with conventional test machines; the method is also applicable to multiaxial and/or non-monotonic tests. We apply this technique to different classes of solids with wide ranges of stiffness, strength and ductility, and discuss the details and the effectiveness of the measurements.

In Section “Materials, specimen preparation and instrumentation” we describe the manufacturing and instrumentation of the specimens used, the test technique is presented in Section “Mechanical stiffness-sensing tests”, results are presented and discussed in Section “Results and discussion”.

## Materials, specimen preparation and instrumentation

The materials to tests were selected based on their ready availability, on the fact that they had been previously characterised by conventional mechanical tests and microscopy in other studies, and on their tendency to develop considerable damage in uniaxial loading.

### CFRP specimens for through-thickness testing

Carbon fibre reinforced polymer (CFRP) specimens for tensile tests in the through-thickness direction were manufactured from a composite laminate of thickness 100 mm, comprising multiple layers of unidirectional carbon fibre laminae with fibre volume fraction of 65%. The laminae comprised HTS-268-1200 high tensile strength carbon fibres of diameter 7 μm and toughened 977-2 epoxy. Relevant mechanical properties of the fibre and epoxy resin are listed in Table 1. The laminate had layup [0/45/-45]_ns_ (with occasional double layers) and ply thickness of 0.3 mm; the material was the same studied in^18,19^.

**Table 1:** Properties of the constituents used to manufacture the CFRP specimens.

	Elastic modulus (GPa)	Transverse elastic modulus (GPa)	Tensile strength (MPa)	Poisson’s ratio
Fibre (HTS-268-1200)	238	35	4240	0.2
Matrix (Epoxy 977-2)	3.52	–	81	0.39

**Figure 1:**
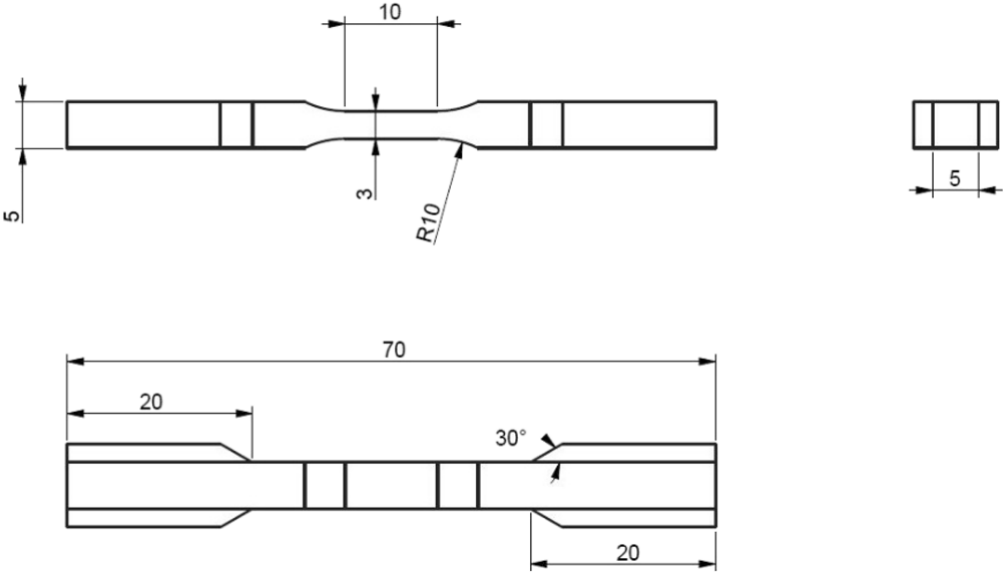
Geometry of the CFRP specimens for through-thickness testing.

Water-jet cutting and grinding were used to manufacture the specimens shown in Fig. 1, with the axis of the specimen coinciding with the through-thickness direction of the composite laminate, such to apply interlaminar tensile stresses during the tension tests.

For the purpose of mechanical testing, the specimens were instrumented with two resistance strain gauges of gauge length 10 mm, applied on opposite sides of the specimen’s gauge portion. This CFRP material was also chosen to perform acoustic and electrical stiffness-sensing tests, to compare the results with those from mechanical stiffness-sensing experiments. In the acoustic/electric tests we simultaneously measure *in-situ* both the speed of sound and the electrical resistance between the specimens ends. The specimens had a different geometry from those in Fig. 1, as they were larger and had a more abrupt transition between ends and gauge portion (the gauge portion measured 10 × 6 × 4 mm with a shoulder radius of 10 mm); this was due to the requirement of a larger cross-sectional area to host the necessary instrumentation, and to limitations of our manufacturing tools. The specimens also had small surface defects originating from water-jet cutting along their shoulder portion, which resulted in them fracturing very close to their ends.

**Figure 2:**
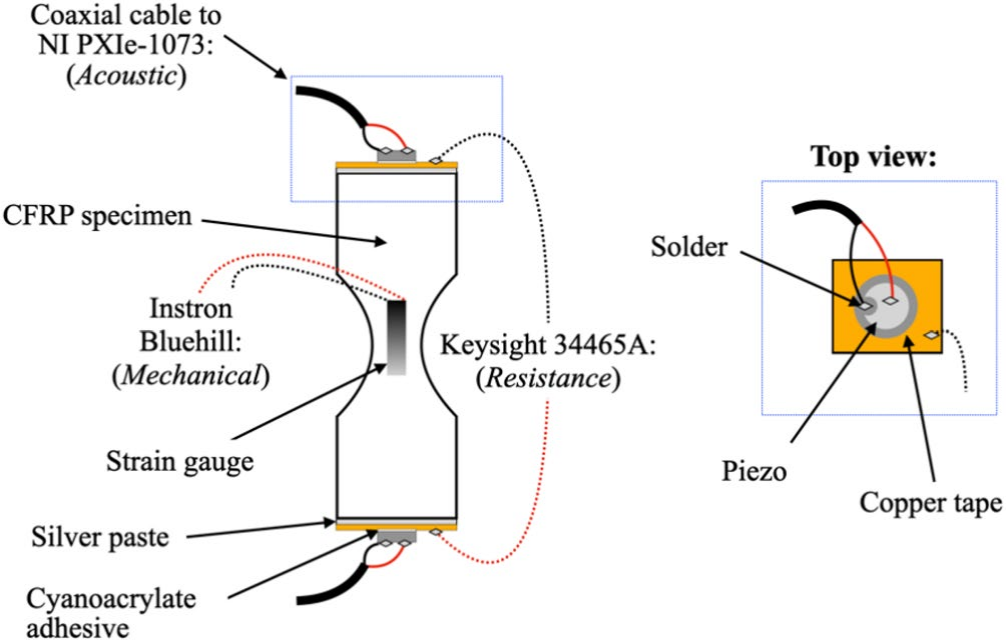
CFRP sample instrumentation for measurements of the speed of sound and electrical resistance.

The specimens carried electrically insulating end-tabs made from a glass fibre composite at the locations in contact with the grips of the test machine, and they were instrumented as sketched in Fig. 2. In brief, a copper foil, to serve as an electrode, was adhered to the specimen’s ends using aluminium paste, and electrical connections were soldered to the copper foil to perform 2-point resistance measurements via a Keysight 34465A digital multimeter, which sampled the specimen’s real part of the electrical impedance at 10 Hz during mechanical loading.

Disc-shaped piezoelectric sensors/actuators (Physik Instrumente PIC255, of diameter 10 mm and thickness 1 mm) were glued with cyanoacrylate on top of the copper tape, and each disc was connected to two wires using pre-manufactured soldering junctions, to provide electrical excitation to one of them (actuator) and read the voltage on the opposite one (sensor). The roles of sensor and actuator were swapped between the two piezoelectric discs at every acquisition cycle (acquisition cycles had frequency of approximately 0.05 Hz). The conditioning was done via a NI PXIe-1073 instrument, housing a NI PXI-451 waveform generator, a Pickering PXI 12 × 8 coaxial matrix for signal actuation and sensing, and a NI PXI-5105 8-channel digitiser for signal acquisition. The excitation was a 5-cycle, 250 kHz sine Hanning window tone burst of amplitude 1 Volt. This procedure, and appropriate interpretation of the measured signals, allowed determining the time delay between emission of a signal by the actuator and arrival of the same acoustic signal at the sensor.

To calculate the resistance of the gauge portion it was assumed that the specimen could be idealised as two resistors in series, one variable, $R_{G}$, corresponding to the gauge portion of the specimen, where substantial and approximately uniform deformation occurs, and one fixed, $R_{E} ,$ corresponding to the tapered ends of the specimen, where deformation is negligible by design. $R_{E}$ also includes any contact resistance and the resistance of the clamped portions of the specimen, the first of which is expected to be negligible in these tests compared to the resistance of the material. The initial resistance of the unstrained specimen was used to determine the initial conductivity of the material. The change in total resistance observed during the tests was interpreted as a change in $R_{G}$ only, and this allowed a trivial calculation of the conductivity of the material at any given strain.

To calculate the speed of sound in the gauge portion of the specimen we observed that the measured time delay $\Delta t$ can be split into the sum of the time taken by the acoustic signal to travel along the gauge section, and the time used to travel along the rest of the specimen, i.e.

2 $$\Delta t = L_{G} /c_{G} + L_{E} /c_{E} ;\quad \Delta t_{0} = \left( {L_{G} + L_{E} } \right)/c_{E}$$

where $L,\,c$ indicate current lengths and speeds of sound, and subscripts *G* and *E* denote gauge portion and specimen’s ends, respectively. The initial speed of sound $c_{E}$ was uniform in the specimen and could be calculated from the initial time delay prior to straining, $\Delta t_{0}$. The stiffness of the specimen $E_{S}$ could be estimated, in first approximation, as

3 $$\sqrt {E_{S} /\left[ {\rho \left( {1 - \nu_{12} \nu_{23} } \right)} \right]} = c_{G} .$$

### Epoxy tensile specimens

**Figure 3:**
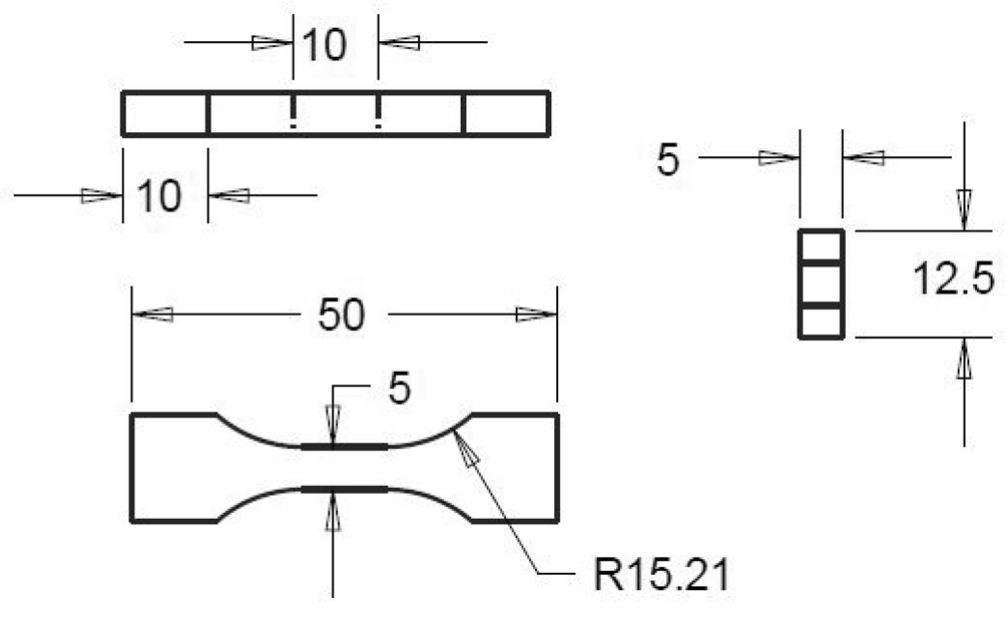
Geometry of the tensile specimens made from the neat epoxy resin.

Dogbone specimens were made from an epoxy resin very similar to that used in the manufacturing of the CFRP laminate, for the purpose of mechanical stiffness-sensing tests. Epoxy plates of thickness $5{\text{ mm}}$ were produced by curing the epoxy resin between two glass panels, as described in^20^. Dogbone specimens were machined by milling of the plates to have a gauge section of $10 \times 5 \times 5\;{\text{mm,}}$ as shown in Fig. 3. The specimen surfaces were polished by hand with sandpaper (down to 2500 grit) to minimise surface defects, and two resistance strain gauges of gauge length 5 mm were adhered to opposite sides of the gauge portion.

### 3D printed and injection-moulded PLA specimens

**Figure 4:**
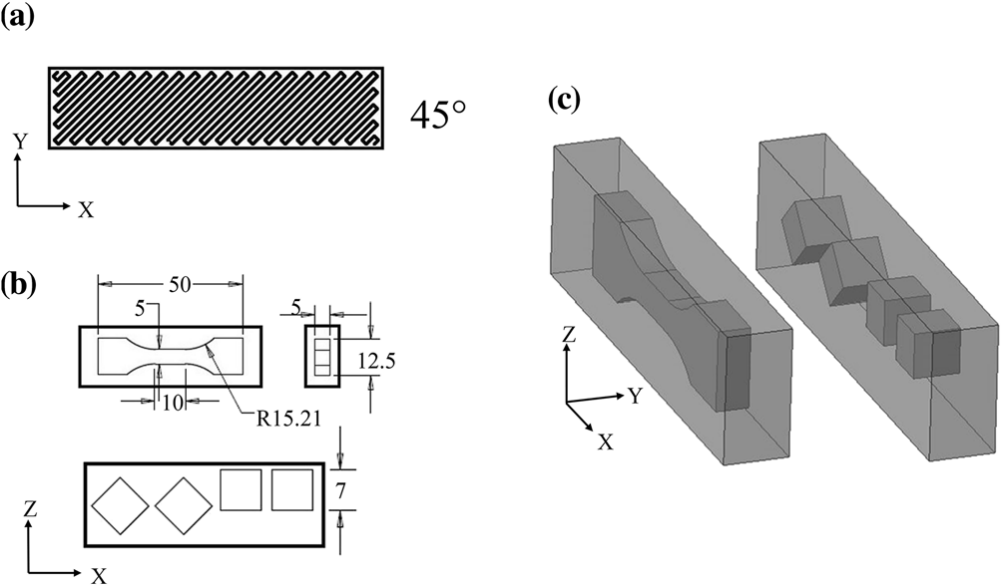
(**a**) Illustration of 3D-printing extrusion in the printed blocks. (**b**) Dimensions of the tensile and compressive specimens. (**c**) 3D-view of the machining scheme.

3D printed PLA specimens were produced using a FDM 3D printer from a single spool of PLA filament procured from IMAKR (http://www.imakr.com). The material was identical to that investigated in^21^, to which we refer the reader for further details. The 3D printer was operated to deposit material in a single direction, resulting in an orthotropic microstructure, with printer settings chosen to obtain a porosity of less than 5%. With reference to Fig. 4, we denote as *XYZ* a global Cartesian reference system in which *X, Y* lie in the plane of each 3D printed layer and *Z* is the out-of-plane direction. PLA blocks (of dimension $70 \times 12 \times 20\;{\text{mm}}$ in the *X*, *Y*, *Z* directions, respectively) were 3D printed, as indicated in Fig. 4a, with extrusion at 45° with respect to the *X* direction. Conventional subtractive manufacturing was used to machine from these blocks both tensile dogbone specimens (with dimensions identical to those in Fig. 3) and cubic compression specimens of side length 7 mm, as shown in Fig. 4b,c. Both uniaxial tension and uniaxial compression tests were performed, such as to load the material at an angle of 45° with respect to the extrusion direction. The choice of this particular angle (rather than, for example, 0° or 90°) was to maximise the evolution of damage, based on the findings in^21^.

Tension and compression specimens were also manufactured from injection-moulded PLA. The same PLA filament used in the 3D printing was injection-moulded using a piston-driven injection moulder (Haake Minijet II, ThermoFisher Scientific, Hampshire, UK) into prismatic blocks, which served as the base material for the manufacturing of the specimens. The barrel temperature and the mould temperature used were 185 °C and 68 °C, respectively; all samples were injected with an injection pressure of 300 bar for an injection time of 20 s, and a pressure of 100 bar was applied for 10 s following the injection. Dogbone specimens were manufactured, with gauge section of dimensions $10 \times 3 \times 3\;{\text{mm}}$, as well as cuboidal compression specimens of dimensions $4 \times 3 \times 3\;{\text{mm}}{.}$ The porosity of the injection-moulded specimens was less than 3%. The injection-moulded specimens were selected for the purpose of comparison to the 3D printed samples, considering that the injection-moulded samples did not have the filamentous nature of 3D printed specimens.

### Sintered Ti powder specimens

**Figure 5:**
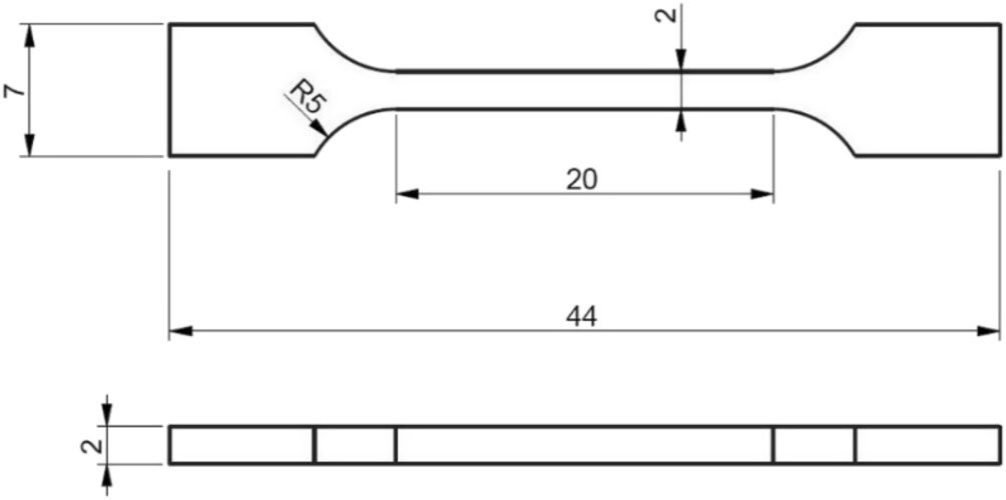
Geometry of the tensile specimens made from sintered Titanium powder.

Dogbone specimens were produced by machining powder-sintered blocks, consisting of a porous metallic solid of porosity around 10%. This material has been extensively described and tested in^22–25^. Sintered Ti blocks were machined into dogbone specimens with gauge section of dimensions $20 \times 2 \times 2\;{\text{mm,}}$ as shown in Fig. 5, using wire electrical discharge machining (EDM). The surfaces of the gauge portion of the specimens were then polished with a buffing wheel to obtain a smooth surface, to which we adhered strain gauges of gauge length 5 mm prior to testing.

## Mechanical stiffness-sensing tests

### Loading histories

Loading of the specimens in both tension and compression was achieved by operating the test machines in displacement control. The history of the imposed displacement was chosen to obtain numerous unloading/reloading cycles in the stress–strain curves, to allow extraction of the current stiffness of the specimens during uniaxial mechanical testing.

**Figure 6:**
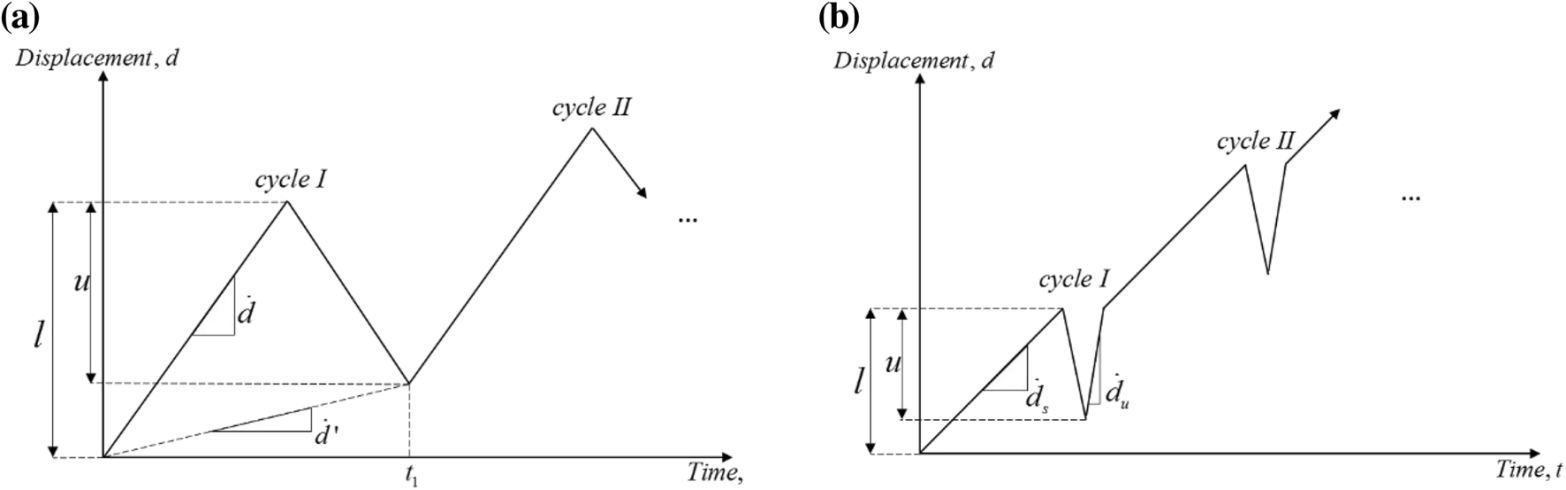
Displacement profiles for elastic modulus measurement. (**a**) First type; (**b**) second type.

Two types of displacement profile were employed, as shown in Fig. 6. The first profile consisted of continuous loading/unloading, comprising a loading displacement ramp of amplitude $l$ followed by an unloading ramp of smaller amplitude $u$, both conducted at equal displacement rate $\dot{d}$. This resulted in an effective (average) displacement rate $\dot{d}^{\prime }$ given by

4 $$\dot{d}^{\prime } = \dot{d}\frac{{\left( {l - u} \right)}}{{\left( {l + u} \right)}}$$

as illustrated in Fig. 6a. Preliminary sets of tests were conducted to guide the choice of the test parameters. This choice was made to ensure a strain amplitude of each unloading cycle of at least 50 με, in consideration of the resolution of the strain acquisition system employed, of approximately 5 με The number of cycles and the test time duration were both dictated by the difference in loading and unloading amplitude, and such difference was chosen to ensure a reasonably short test time, to minimise stress relaxation, and to obtain a sufficiently high number of cycles.

The displacement history in Fig. 6a was found adequate for all materials tested, with the exception of the sintered Ti powder. For this material the specimens were found to be affected by cyclic loading creep. Understanding the reasons for this behaviour (whether it was driven by dislocation plasticity, by internal frictional contact between powder particles or by other mechanisms) are beyond the scope of the present study; this response resulted in a delay between the unloading imposed by the test machine and the change in sign of the strain rate. This, in turn, distorted the unloading/reloading cycles and made determination of the current stiffness difficult. A modified loading profile was therefore employed for the Ti specimens, as shown in Fig. 6b. This consisted of a loading ramp of amplitude $l$ with rate $\dot{d}_{s}$, followed by much faster unloading/reloading ramps of amplitude $u$ and rate $\left| {\dot{d}_{u} } \right|$ in both unloading and reloading. This different cycle resulted in a smaller number of unloading/reloading cycles, however the stress–strain histories recorded in these cycles were much less affected by cyclic loading creep and allowed to easily deduce the current specimen stiffness.

We note that in both types of tests the strain rate during the unloading and reloading phase were equal, in consideration of the relatively small change in stiffness of the specimen, which will be shown below. In tests of the first type, the magnitude of the strain rate was the same in all phases of the tests, which is particularly important when testing polymeric materials. An example of the displacement and strain histories recorded during a test on an Epoxy resin are provided in the Appendix (Supplementary Material).

### Instrumentation and loading devices

All tests were conducted on an Instron universal tensometer (model 5969) equipped with a 50 kN resistive load cell. Flat V-shaped clamps were used to grip the ends of the dogbone specimens, while compression specimens were loaded by polished, parallel steel platens, lubricated by PTFE spray. The required displacement histories were achieved by custom loading profiles coded in the BlueHill control software of the Instron machine.

Accurate measurements of the small surface strains during the unloading/reloading cycles were made by resistance strain gauges (Tokyo Sokki Kenkyujo Co. Ltd.) as detailed in Table 2, which also reports the applied displacement amplitude and rates, which were chosen to obtain average strain rates between $10^{ - 4} \,{\text{s}}^{ - 1}$ and $10^{ - 2} \,{\text{s}}^{ - 1}$, as recommended in ASTM standards for metals and plastics (ASTM E8/E8M-16a and ASTM D638-14, respectively). The strain reading system comprised an integrated Fylde Wheatstone bridge and amplifier, operated in quarter-bridge mode, with an excitation voltage of 1 V, a gain of 500× and full bandwidth. All electrical junctions were soldered, which improved notably the strain measurement resolution. A laser extensometer of resolution 1 μm was also used to measure the elongation of the gauge portion and confirm the average strain in the gauge section.

**Table 2:** Strain gauges and test parameters used for different materials.

Material	Strain gauge	Loading amplitude $l$ (mm)	Unloading amplitude, $u$ (mm)	Unloading rate, $\dot{d}$ (mm/s)	Loading rate, $\dot{d}^{{\prime }}$ (mm/s)	Average strain rate (/s)	Unloading/ reloading strain rate (/s)
CFRP	FLKB-10-23	6.0 × 10^–3^	5.5 × 10^–3^	1.0 × 10^–3^	4.3 × 10^–5^	4.1 × 10^–6^	± 2.2 × 10^–4^
Epoxy	FLA-5-23	6.0 × 10^–3^	5.5 × 10^–3^	1.0 × 10^–3^	4.3 × 10^–5^	4.5 × 10^–6^	± 2.2 × 10^–4^
PLA (Tension)	FLA-5-50	6.0 × 10^–3^	5.5 × 10^–3^	1.0 × 10^–3^	4.3 × 10^–5^	8.2 × 10^–6^	± 2.7 × 10^–4^
PLA (Compression)	FLK-1-23	4.3 × 10^–3^	3.8 × 10^–3^	7.0 × 10^–4^	3.5 × 10^–5^	2.0 × 10^–5^	± 7.3 × 10^–4^
Sintered Ti powder	FLA-5-23	3.0 × 10^–2^	2.0 × 10^–2^	$\left| {\dot{d}_{u} } \right| = {1}.0 \times {1}0^{{ - {2}}}$	$\dot{d}_{s} = {1}.0 \times {1}0^{{ - {3}}}$	3.1 × 10^–5^	± 3.2 × 10^–4^

### Data processing

**Figure 7:**
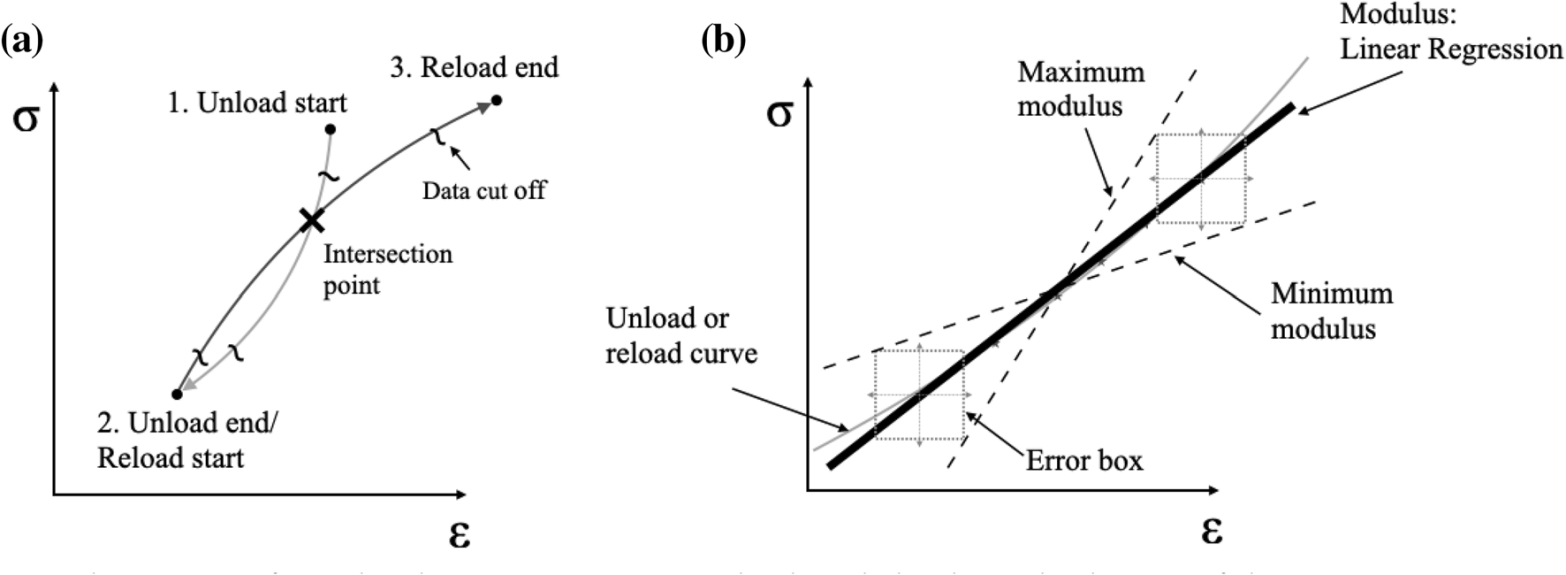
Schematics of (**a**) the data processing method and (**b**) the calculation of the uncertainty in stiffness measurements.

Unloading/reloading cycles showed in most cases the features sketched in Fig. 7a. Points 1, 2, 3 in Fig. 7a were identified for each cycle, splitting the stress versus strain data into an unloading and a reloading branch; a number of datapoints equal to 15% of the total was discarded at the beginning and the end of each branch; the remaining 70% of the datapoints was used to perform a least-square fit of the data to a linear function, providing an unloading and a reloading modulus. The number of datapoints used to perform such data-fittings was recorded at every cycle. In all experiments the unloading modulus was found to be higher than the loading modulus, as expected.

An uncertainty in the measurements of such unloading and reloading moduli was calculated based on the uncertainties associated to the force and strain measurements, which were taken as the average noise in the data and were of ± 0.2 N and ± 2.0 × 10^–6^ for the force and strain, respectively. Figure 7b shows schematically how the uncertainty in the moduli was calculated. This was based on identifying the extreme points of each unloading and reloading branches of the cycle included in the data-fitting, and performing the geometric construction in Fig. 7b to estimate the maximum and minimum possible modulus (note that the boxes shown in Fig. 7b represent schematically uncertainty in true stress and true strain measurements).

In most cases, the unloading and reloading branches of the stress strain curves in each cycle formed loops, as sketched in Fig. 7a. The area enclosed in each loop, as well as the area under the reloading portion of each loop were also recorded, and their ratio was used to quantify the viscous response of the solid. We describe this procedure in more detail and show preliminary results in Appendix A.

## Results and discussion

In the following we present and discuss the results from selected experiments conducted on each material. For each test we show the measured true stress versus strain curve as well as the corresponding evolution of unloading and reloading moduli as a function of the applied strain. Details of the unloading/reloading cycles are provided at different stages of the measured response, to illustrate the quality of the data used to measure the stiffness evolution. For some experiments the measured stress–strain curves in stiffness-sensing tests are compared to curves measured in simple monotonic tests, conducted at the same average strain rate to rule-out any bias caused by strain rate sensitivity of the materials tested.

### CFRP and neat epoxy

**Figure 8:**
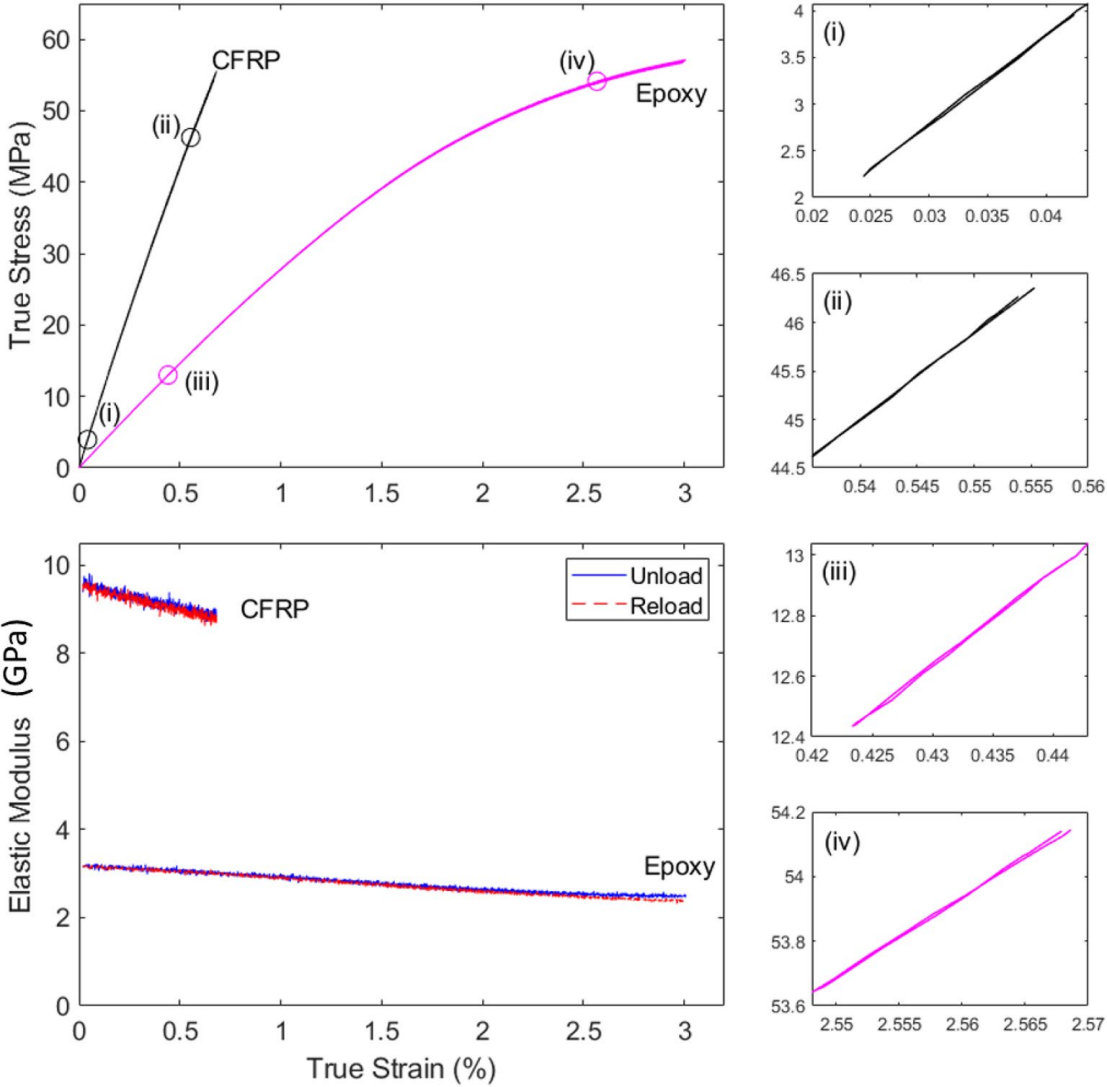
True stress versus strain and stiffness (true elastic modulus) versus strain curves measured for the CFRP and the neat epoxy specimens.

The stress–strain curves presented in Fig. 8 for the CFRP specimens and the neat epoxy are in line with the monotonic measurements published in^18–20^, indicating that the unloading/reloading cycles imposed in this study do not notably alter the measured stress–strain response of the materials. As expected, the CFRP is stiffer but slightly weaker than the neat epoxy, due to the presence of the carbon fibres (we recall that the matrix used in the manufacturing of the CFRP is very similar to the neat resin tested in this study). The details of the unloading/reloading cycles shown in Fig. 8 show a low degree of hysteresis at the early stages of macroscopic deformation, increasing for the case of the epoxy at large strains, in line with the increase in the difference between the measured unloading and reloading moduli with increased strain.

The stiffness measurements show, for both materials, a steady degradation of the elastic properties with increased strain. Such degradation is more pronounced for the CFRP (~ 5% decrease in modulus at a strain of 0.5%) than for the epoxy (~ 3% decrease at the same strain). This is plausible, in consideration of the strain concentrations induced by the carbon fibres.

It is particularly noteworthy that we do not observe a pronounced reduction of the stiffness of the specimens prior to their final fracture. This indicates that this method is unable to capture the final localisation of damage in the tension tests. Both materials exhibited brittle fracture, consistent with the notion that at the final stages of the tests damage localised in a relatively small volume of the specimen, such that the corresponding local reduction in stiffness was too small to affect the global specimen stiffness considerably (we recall that the strain gauges covered the entire length of the specimen’s gauge portion in both cases, and that this choice was intentional, to obtain average measurements over the gauge length). This suggests that the measured stiffness degradation must indicate an approximately uniform growth of damage in the solid, prior to localisation.

Such uniform damage growth begins at the very early stages of the deformation for the CFRP. This has considerable implications in the modelling choices for these types of solids. Researchers currently confidently assume that the response of a CFRP is linear elastic up to stresses of the same order of the measured failure stress; our more detailed measurements show that this is not the case: the response of the material is not linear, while it may look linear after a superficial analysis; damage increases uniformly since very small values of the applied macroscopic strain and the stiffness-sensing technique offers a quantitative insight into such damage evolution. Similar considerations hold for the epoxy resin: typical approaches to constitutive modelling of this material would assume, based on the shape of the curve, a damage initiation strain well above the so-called macroscopic yield strain of the material. Our measurements show that damage initiates much earlier than this; for example, at a strain of 0.5% the measured stiffness has already reduced significantly, while the specimen dimensions have not, and the material is still experiencing what would currently be considered a macroscopically viscoelastic response. This suggests that a new class of constitutive models needs to be developed for ductile solids, allowing for the possible early occurrence of local damage prior to bulk plasticity.

**Figure 9:**
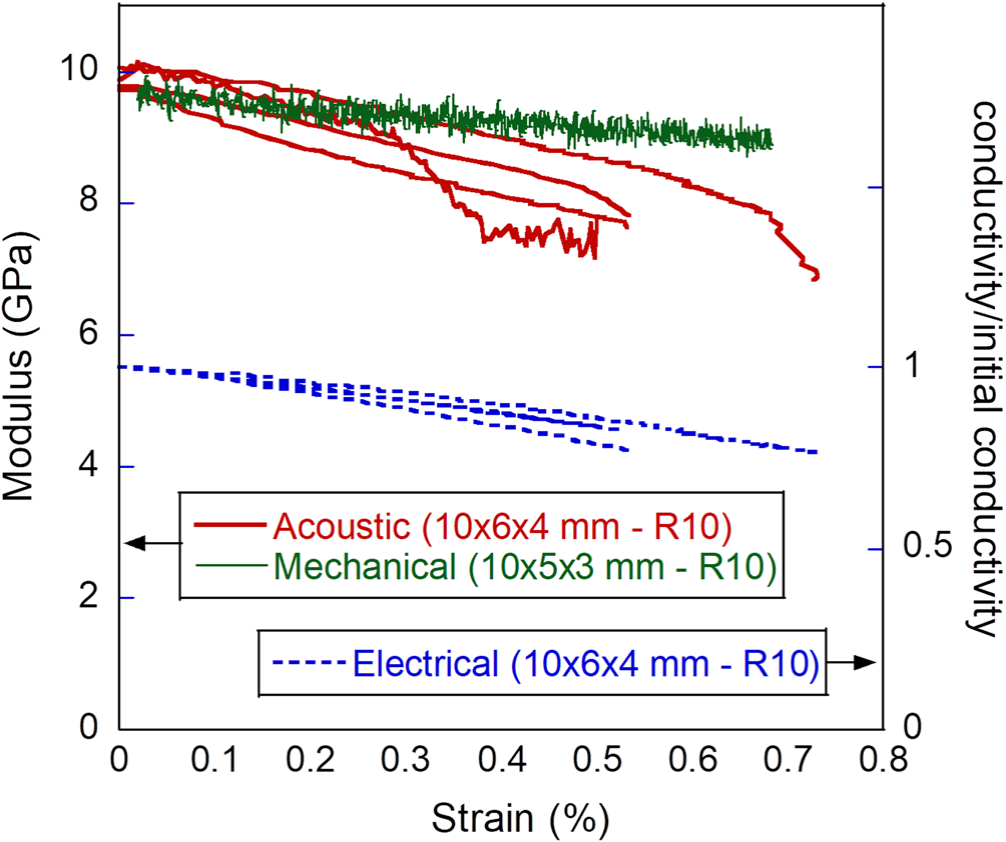
True strain versus mechanical and acoustic modulus, and relative conductivity for CFRP specimens.

To reinforce the notion that the measured degradation in specimen stiffness is the consequence of a physical phenomenon, rather than an artifact from the measurements and data processing, additional stiffness-sensing tests were conducted on the CFRP samples, instrumented as described above. In these additional tests the loading was monotonic, but they included simultaneous measurements of the speed of sound along the axis of the specimen as well as the conductivity of the gauge portion of the specimen. Figure 9 presents the calculated specimen’s stiffness based on measurements of the speed of sound in the material, and these are compared to the results of the mechanical stiffness-sensing tests presented in Fig. 8. The two sets are in broad agreement: they start at the same initial modulus for the unstrained material and both show a monotonic decrease in modulus with applied strain. The tests on the larger specimens show a more pronounced decrease of the modulus with strain, possibly due to the different volume of the specimens, possibly to the manufacturing defects in the larger specimens. In any case, the acoustic measurements confirm that the speed of sound is decreasing in the material; in consideration of the fact that the density does not change substantially in these tests, this must indicate a decrease in stiffness.

Figure 9 also shows the evolution of the material’s conductivity as a function of strain, showing a monotonic decrease with strain, consistent with the notion that damage and loss of cohesion is developing in the material. These are clear indications that the decrease in stiffness with strain measured in the mechanical stiffness-sensing tests quantifies the physical response of the system and is unlikely to be an artifact of our data-processing.

We note here that the reduction in specimen’s stiffness recorded with the proposed method is much higher than what can be ascribed to geometric effects. This is discussed further in the Supplementary material (Appendix A.2).

### Injection-moulded and 3D printed PLA

**Figure 10:**
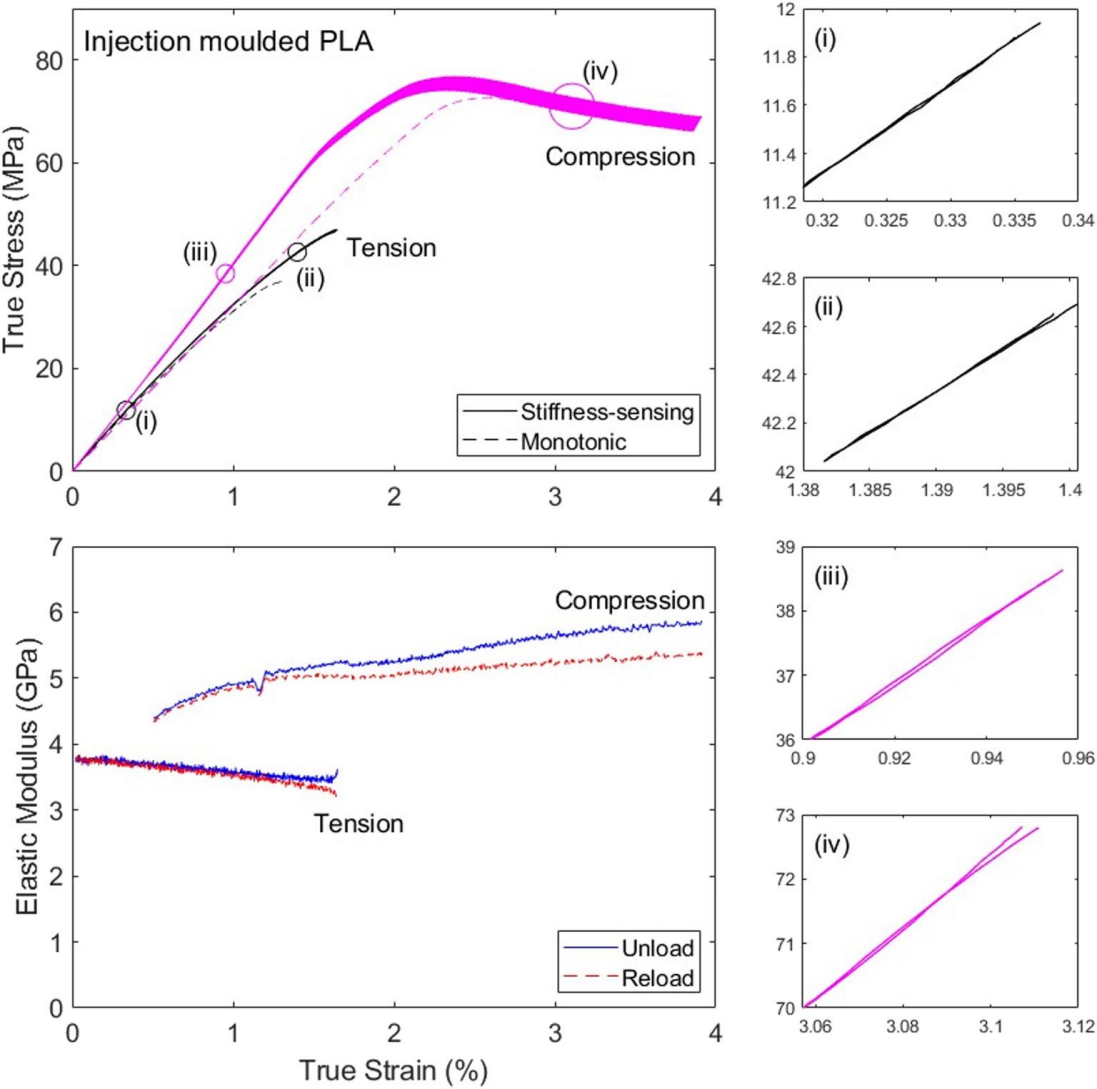
True stress versus strain and stiffness (true elastic modulus) versus strain curves measured for the injection-moulded PLA in both tension and compression.

**Figure 11:**
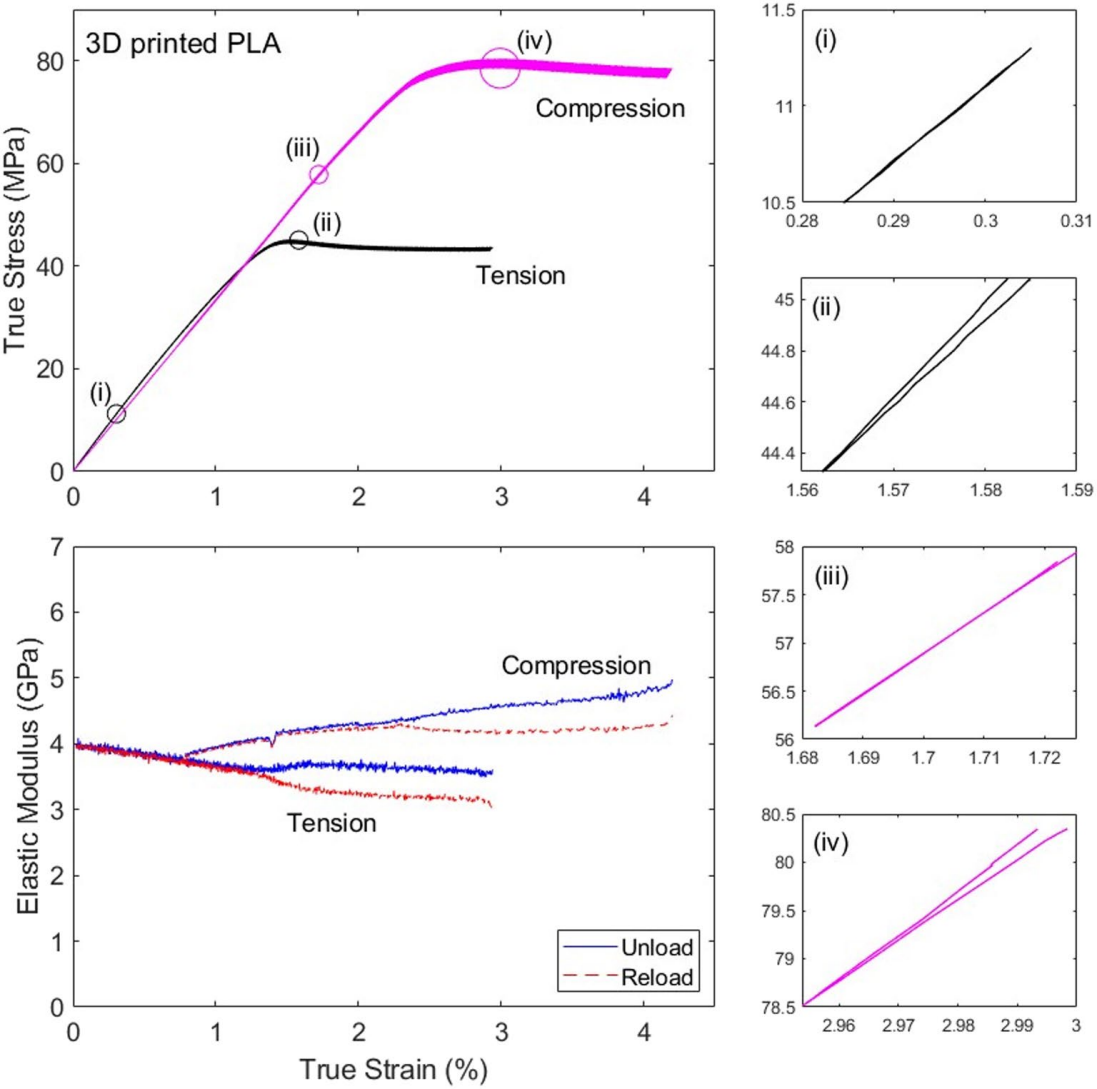
True stress versus strain and stiffness (true elastic modulus) versus strain curves measured for the 3D printed PLA (loaded at $45^\circ$ with respect to the extrusion direction) in both tension and compression.

Figures 10 and 11 present data for the injection-moulded and 3D-printed PLA, respectively. The stiffness-sensing protocol used in the current study did not affect considerably the measured stress–strain responses of the material in tension and compression, compared to the measurements reported in^21^. A direct comparison between monotonic and stiffness-sensing tests (at the same average strain rate) is presented in Fig. 10 for the injection-moulded material; note that the discrepancy in the measured stiffness and strength, particularly noticeable in compression, is comparable to the level of scatter in material properties reported in^21^.

We note that for both materials the specimens failed in tension by brittle fracture, while the compressive response was ductile and tests were interrupted at a strain of approximately 4%. The dataset for the tension test on the 3D-printed material in Fig. 11 is incomplete, due to premature failure of the strain gauges at approximately 3% strain (the portion of stress–strain curve at strains exceeding 3% was completed using data from the laser extensometer). For both materials the compressive stiffness versus strain data at strains below 0.5% is not presented, for the sake of clarity. This is due to the fact that the data showed high noise and an apparent pronounced stiffening with increasing strains, likely due to bedding-in effects (we recall that small cuboidal specimens were used in compression).

We recall that both materials have some degree of porosity. While in tension the measured stiffness decreases with increasing strain, in compression the opposite is observed. This is in line with the notion that pores are being closed in compression, while they tend to expand in tension; similar conclusions were shown for a different material in^25^, supported by microscopy observations. We showed in^21^ that the microstructures of the two materials were very different: while the injection-moulded specimens were homogeneous, and had porosity of order 3%, the 3D-printed material had a filamentous microstructure and higher porosity (5%). This resulted in very different failure mechanisms for the two materials, despite their mechanical properties and stress versus strain curves were comparable. In this study indeed we measure similar stress–strain curves for the two materials, but substantially different modulus versus strain curves, indicating that the proposed stiffness-sensing technique can enrich the measurements performed in mechanical tests. Specifically, in tension the injection-moulded PLA shows a less rapid stiffness degradation than the 3D printed PLA, indicating a higher damage for the 3D printed material than for the injection-moulded one (at the same strain). In compression, the stiffness increases with strain less rapidly for the injection-moulded specimens than for the 3D printed material, consistent with the fact that the former has less porosity than the latter.

We note that for the 3D printed material in Fig. 11, at sufficiently large strains and in both tension and compression, the unloading and reloading moduli start to diverge. This is a consequence of the fact that the stress versus strain histories during unloading/reloading cycles change shape, as shown in the insets of Fig. 11; in particular, loops are no longer formed, or if they form, they are small compared to the extent of the unloading/reloading cycles. Our confidence in the physical significance of our measurements in this regime is low, however the data is still presented in Fig. 11 to illustrate this possible limitation of the technique. This problem could possibly be avoided by limiting the analysis of stiffness to only the loops in the stress–strain curve, or using a different loading profile, however this is not pursued here and left as a topic for future investigations.

We also note that the comparison between tensile and compressive responses is unfair, as different specimen geometry and different length of strain gauge grids were used in the two types of tests, and the evolution of the macroscopic stiffness is expected to be specimen-dependent. Tests conducted on cuboidal specimens may also have been affected by a non-uniform distribution of stresses and strains, which might alter the accuracy of the stiffness measurements. In compressive the tests on PLA shown in Figs. 10 and 11, we measured an increase in stiffness of order 15–20%, in the portion of the data showing similar measures of the unloading and reloading modulus. This is too high to depend exclusively on the reduction of porosity, which was of order 5% (a Voigt upper bound would suggest the material’s stiffness should increase by up to 5% when all pores are closed). This inconsistency cannot be attributed to geometric effects, which are negligible at the strains considered (up to 1% and 2% for injection-moulded and 3D-printed PLA, respectively), as shown in Appendix A.2. This could be partly due to an intrinsic increase in stiffness of the polymeric material, by a strain-induced re-alignment of the polymeric chains; it might be also due to the presence of a higher porosity than what we measured using optical methods, however we do not possess sufficient evidence to support these claims. Measurements in compression should be repeated with identical specimen geometry and instrumentation as in the tensile tests to enable a more meaningful comparison of the measurements in tension and compression.

### Sintered Ti powder

**Figure 12:**
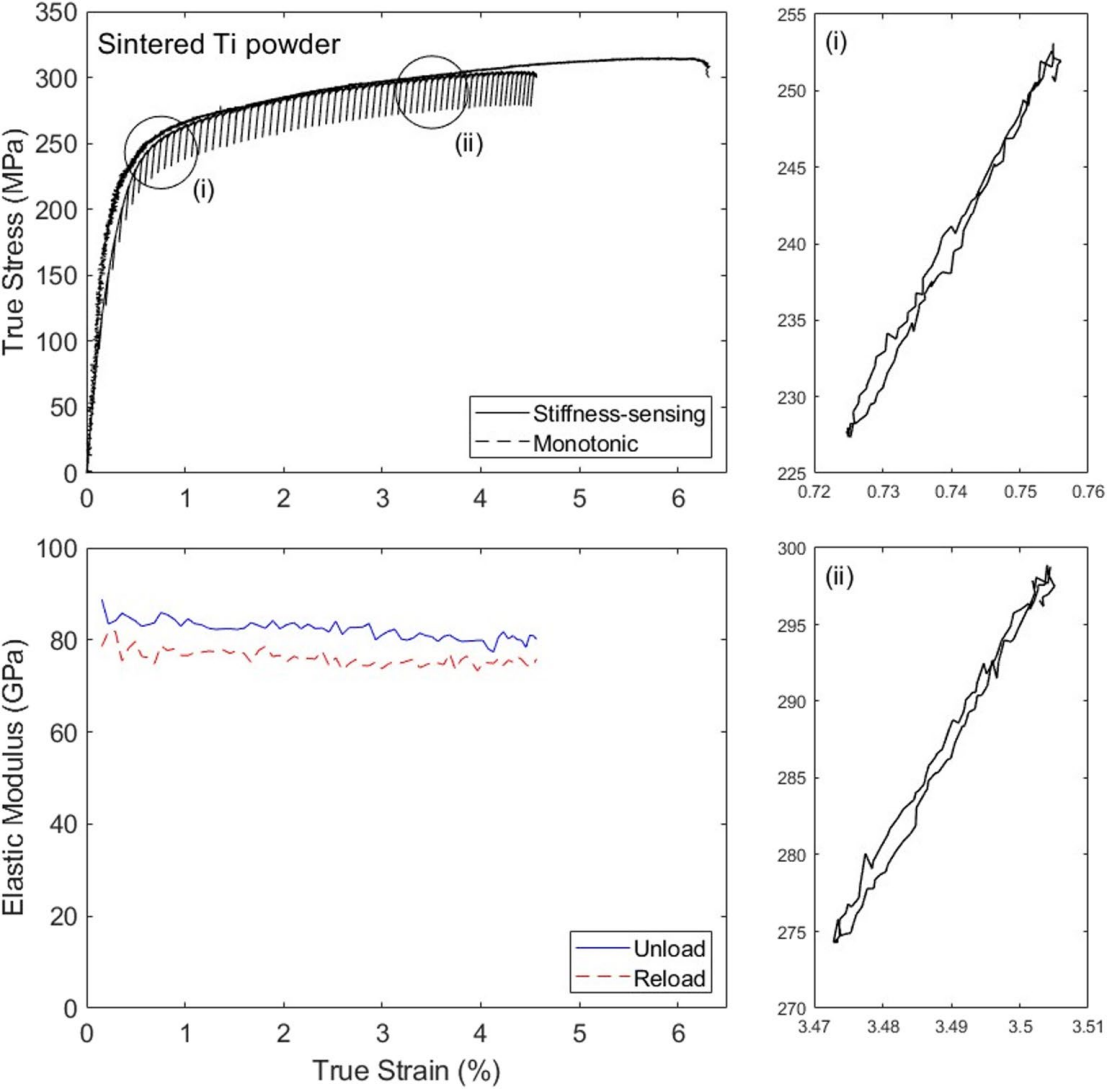
True stress versus strain and stiffness versus strain curves measured for the sintered Ti powders.

Representative results for the sintered Ti specimen are presented in Fig. 12. The stress–strain curve measured in a stiffness-sensing test is compared to that measured in a monotonic experiment on an identical specimen, and the two are found in broad agreement, with a difference comparable to the scatter displayed in the response of this material in our previous studies. We note that due to the high stiffness of the material, the stress amplitude of the unloading/reloading cycles is higher for this solid than for the other materials tested in this paper (while the strain amplitudes are comparable for all tests).

This porous solid was tested using a displacement profile of the second type (as described in Section “Loading histories”); it consequently displays a smaller number of unloading/reloading cycles compared to the other materials tested. Despite the less ‘continuous’ and more noisy measurements of the stiffness, even in this case the data supports the notion of a decrease in stiffness beginning at the early stages of deformation. Note that in this test the specimen failed just outside the strain gauge grid, which in this case did not cover the entire gauge portion. The unloading modulus was found to be larger than the reloading modulus, suggesting some degree of energy dissipation during the unloading/reloading cycles. Microscopic observations conducted in^25^ before and after testing of this material showed evidence of pore cavitation induced by the uniaxial tension, and the effects of this are captured by the mechanical stiffness-sensing proposed here. Again, the results suggest that a constitutive model allowing the simultaneous development of damage and local plastic strain at small strains should be employed to model effectively the material response.

### Limitations of the technique and possible improvements and applications

The design of the experiments shown in this study involves a careful balance of unloading/reloading strain amplitudes and rates, number of unloading/reloading loading cycles, and resolution of the strain reading. Ideally the amplitudes of unloading/reloading cycles should be minimised while their rates and the number of cycles should be maximised, to ensure near-continuous measurement of the stiffness, obtain a less invasive test technique and minimising any measurement artefacts related to time-dependent response of the materials. Table 3 lists, for each of the tests presented here, information on the number of datapoints used to determine the unloading and reloading modulus, as well as estimates of the uncertainty on the measured unloading and reloading stiffness. The number of points used to determine the stiffness is adequate, but an increase in such number would be beneficial and would lead to smoother stiffness measurements. The uncertainty in stiffness is considerably less than the measured variations with strain.

**Table 3:** Number of datapoints used to determine the unloading and reloading modulus for all materials tested. Uncertainty on the unloading and reloading stiffness.

Material	N. of datapoints used (min–max)		Average uncertainty in stiffness (%)	
	Unload	Reload	Unload	Reload
CFRP	10–11	10–11	4.45	4.09
Epoxy	10–12	9–11	6.52	5.28
PLA inj. moulded (tens.)	15–20	14–20	5.36	4.87
PLA inj. moulded (comp.)	14–18	15–18	1.28	1.16
PLA 3D (tens.)	13–15	12–15	5.28	4.36
PLA 3D (comp.)	8–10	9–10	1.99	1.49
Sintered Ti powder	37–62	40–66	2.23	1.98

The experiments in this study were conducted in a screw-driven tensometer, which was limited in the precision of the achievable cross-head displacement and in the rate of such displacement. Hydraulic or electro-pulse test machines are expected to be less limited in displacement rate but are inherently operated in load control, which combined with the inertia of their parts might result in problematic and less precise displacement histories. A possible solution to these problems could consist of connecting a dedicated stiffness-sensing actuator in series with the specimen, to apply unloading/reloading displacements to superimpose to the monotonic displacement of the machine’s cross-head. As the unloading/reloading amplitudes are to be kept small (recall Table 1), piezoelectric actuators would be ideal candidates for this task, due to their inherent displacement control, strength, speed and ease of actuation.

The strains associated with the unloading/reloading cycles necessary for this technique are small, and their accurate measurement is very important to obtain adequate stiffness-sensing. This makes the application of DIC techniques hard, in consideration of the fact that the entire gauge portion of the specimen needs to be monitored, and that in the case of DIC microscopy, the specimen can come in and out of focus periodically. Resistance strain gauges were used in this study to limit the cost of the tests. 2-point laser interferometry would allow resolving displacements of order of nm, however it would complicate the tests and increase the cost considerably. Mechanical clip gauges might possess sufficient resolution; an additional potentially viable option could be the use of semiconductor wire gauges.

Employing better loading devices (e.g. piezoelectric actuators) and strain diagnostics (e.g. semiconductor gauges) would allow smaller, faster unloading/reloading cycles. The data processing could be further enhanced by driving the actuator at specific frequencies and using Fourier analysis to filter the strain measurement data, as done successfully for indentation tests in Oliver and Pharr^14^. Extension of the technique to biaxial, triaxial and non-monotonic tests would be relatively straightforward. The development of acoustic and resistive measurements such as those shown for the CFRP specimens should also be pursued, as it could provide benchmark data for the mechanical stiffness-sensing tests or replace the mechanical technique, considering the fact that such acoustic and resistive techniques are less invasive for the material, have relatively simple instrumentation and do not limit the test strain rate, and therefore could be applied also in the case of dynamic measurements of the response of solids.

The data obtained from the tests presented here are representative, for a well-designed test specimen, of the effective stiffness of the gauge portion of the samples tested. The evolution of damage in solids however varies in space, and therefore the stiffness-sensing data should be interpreted within a stochastic modelling framework, to be useful in numerical simulations.

In our future studies we shall explore possible improvements to the experimental technique and the corresponding new modelling strategies that such technique enables and requires.

## Conclusions

We have shown the feasibility of a mechanical, near-continuous stiffness-sensing test protocol and applied it to a set of selected materials with wide ranges of mechanical properties. The technique was implemented using standard test machines and strain diagnostics, but suggestions for its improvement were made.

We showed that the proposed stiffness-sensing technique considerably enriches the datasets produced in quasi-static mechanical tests and that the additional data produced are representative of the physical response of the solids tested. In particular the test technique is able to make measurements of the initiation and evolution of damage in solids, providing information that can be used to calibrate constitutive models and that lends itself to be used in the formulation of new data-driven constitutive models.

The measurements presented showed that, for the relatively brittle solids tested in this study, damage initiates at the very early stages of the material response, at very low strains; this is in contrast to what is typically assumed in current numerical damage modelling approaches. This suggests that the development of stiffness-sensing test techniques may have considerable impact in the mechanics of solids and in the formulation of constitutive models for the fracture response of engineering materials.

## Supplementary Information

Supplementary Information.
