# Supplementary material for: A new stiffness-sensing test to measure damage evolution in solids
Source: Sci Rep. 2022 Jan 10;12:472. doi: 10.1038/s41598-021-04452-9 (PMC8749001; doi:10.1038/s41598-021-04452-9)
Supplement: Supplementary file 1 — Supplementary Information. [file 41598_2021_4452_MOESM1_ESM.docx]

**Supplementary material**

**APPENDIX**

**A.1 Example of the displacement and strain histories**

Figure A1 presents an example of the displacement and axial strain histories measured during the test on an Epoxy resin shown in Fig. 8. Despite the small magnitude of the unloading/reloading displacement amplitude, the screw-driven machine imposes a displacement history very close to the desired linear one. The corresponding strain in the axial direction has similar trends and is in-phase with the applied displacement history.

**A.2 Geometric effects in stiffness evolution**

The variation of the specimen’s dimensions during loading introduces a loss of stiffness in tension and a gain of stiffness in compression. To understand the importance of this effect on the measured decrease in stiffness, we conducted a Finite Element simulation of the tensile response of a cuboidal domain of geometry identical to the gauge portion of the Epoxy specimen. Dirichlet boundary conditions were applied, imposing a displacement history mimicking the experiments in this paper. The simulations accounted for geometric non-linearity, but the material was modelled as an isotropic linear elastic solid, of constant, uniform stiffness equal to the measured initial stiffness of the epoxy specimens. The specimen was subject to repeated unloading-reloading cycles, and the slope of the stress-strain curve was used to obtain both the nominal and true Young’s modulus at each unloading cycle. The stiffness of the specimen (in kN/mm) was also calculated and plotted as a function of true strain.

*Figure A1. Displacement and axial strain histories recorded during a test on the Epoxy resin.*

*Figure A2. Displacement and axial strain histories recorded during a test on the Epoxy resin.*

Figure A2 shows that the stiffness of the specimen decreases with increasing tensile strain, as expected. The nominal Young’s modulus (defined as the slope of the nominal stress-strain curve during unloading) also follows the same trend, being proportional to the stiffness. We note however that the true Young’s modulus, which is what we extract from the experiments in this paper to quantify the material’s stiffness, shows a much milder decrease, of negligible extent when compared to what we measure for the epoxy material tested (measurements are included in Fig. A2); this reassures us that we must be measuring the intrinsic degradation of the material’s stiffness, rather than an artifact due to geometry.

With regards to the acoustic and resistive techniques, the measurements were corrected for the change in geometry. The time of arrival of the acoustic signal was used in conjunction with the current length of the specimen to calculate the current speed of sound. The change in density was also accounted for.

**A.3 Quantification of viscous dissipation during the unloading/reloading cycles**

The areas under the unloading and reloading branches of the stress-strain curves in each unloading/reloading cycle were calculated by integration. Where multiple loops were present, due to the unloading and reloading curves being very close to each other, all loops were considered, integrating between the transition point between unloading and reloading (denoted as point 2 in Fig. 7a, and having stress $\sigma_{\min}$) and the intersection point between unloading and reloading branches, having the highest stress. If no loops were formed, the particular unloading/reloading cycle was discarded. A dissipation factor was calculated as the ratio of the area enclosed in each loop, normalised dividing by the area under the reloading area, after subtracting from this the area of the rectangle $\sigma_{\min}\Delta\varepsilon_{loop}$, to make this factor as independent as possible from the current stress.

The dissipation factor is plotted in Figure A3 as a function of strain for all materials tested in this study, after filtering the data by applying a moving average with averaging window width equal to 5% of the total number of datapoints. In most cases this factor tends to increase monotonically with strain and it appears correlated with the difference between the measured unloading and reloading stiffnesses. We recall that in some tests such difference in stiffnesses is likely to be associated to the low quality of the stress-strain data (for example for the tests on the 3D printed PLA at high strains); consequently our confidence in these particular measurements is low. The dissipation factor cannot be considered a material property, because it depends on the strain and stress amplitudes associated with each loop. However it can be related to material properties upon assuming a certain viscoelastic constitutive response. While the results in Fig.  A3 are preliminary, they further confirm that the proposed technique can enrich mechanical measurements with relatively little cost and efforts.

*Figure*  *A3. True strain versus dissipation factor for all specimens.*
